# “Just Stay Home and Wait It Out”: Exploring Challenges Accessing Care for Miscarriage in Indiana

**DOI:** 10.1111/psrh.70057

**Published:** 2026-01-26

**Authors:** Kathryn J. LaRoche, Anayra Maldonado Quiles, Oluwapamimo J. Fafowora, Fatimah Lawal

**Affiliations:** ^1^ Department of Public Health Purdue University West Lafayette Indiana USA; ^2^ Department of Anthropology Purdue University West Lafayette Indiana USA

## Abstract

**Introduction:**

Qualitative research on miscarriage in the United States primarily draws on the experiences of participants recruited from healthcare settings which may fail to robustly illuminate how people navigate to care. We aimed to explore the miscarriage experiences of Indiana residents, identify barriers to access, and generate recommendations to improve comprehensive miscarriage management and information across the state.

**Methodology:**

We used community‐based recruitment methods and conducted semi‐structured, in‐depth interviews with 26 participants who had experienced at least one miscarriage in Indiana between 2018 and 2023. We audio‐recorded and transcribed the interviews, and then we used Dedoose to manage our data and carried out content and thematic analysis.

**Results:**

Participants described significant challenges locating both information and care about miscarriage. When they reached out to health care providers, women were instructed to “wait it out” or go to the emergency department (ED). These recommendations were inconsistent with participants' needs and desires. Participants reflected negatively on their experience at the ED, emphasizing the high financial and emotional costs associated with the visit. Only participants who discovered their pregnancy loss during prenatal care appointments reported being offered active intervention.

**Discussion:**

Our findings suggest that some women in Indiana lack access to information and comprehensive treatment options for miscarriage. This funnels non‐emergent patients to the ED where patients incur significant financial and emotional costs. Developing strategies to support miscarrying women inside and outside of the ED appears warranted.

## 1 Introduction

Between 10% and 20% of known pregnancies in the United States end in miscarriage, defined as the spontaneous loss of a viable pregnancy before 20 weeks' gestation [1, 2]. Miscarriage in the first trimester, also referred to as early pregnancy loss (EPL), accounts for 80% of cases [1] and is the most common pregnancy complication. Patients' perceived quality of medical care and interactions with healthcare professionals (HCPs) during the early stages of miscarriage treatment are crucial in influencing ongoing health outcomes [3]. Additionally, satisfaction with care after a miscarriage may help alleviate symptoms of depression, anxiety, and perinatal grief [3, 4, 5]. However, a global body of evidence has found that women^1^ frequently report dissatisfaction with the miscarriage care they receive and that simultaneously, HCPs feel underequipped to meet the emotional needs of patients [3, 5, 6, 7].

The American College of Obstetricians and Gynecologists (ACOG) lists three treatment options for miscarriage, including expectant management, medication management, or surgical evacuation [1]. Expectant management, or watchful waiting, allows the body time to expel the products of conception without additional intervention and is only recommended in the first trimester. As with induced abortion, medication management uses either misoprostol alone or a combination of mifepristone and misoprostol up to 70 days [8], while surgical evacuation involves the removal of pregnancy tissue from the uterus through aspiration or dilation and curettage (D&C) [1]. There is no difference in the rate of infection between expectant, medication, or surgical management [9, 10, 11], meaning that patient preferences can and should be accommodated when deciding on a treatment plan [1, 12]. A variety of individualized factors influence women's decision‐making about treatment for miscarriage, such as scheduling and availability, anticipated pain, cost, concerns for future fertility, and more [13, 14]. Still, the evidence is clear: women value being offered options outside of expectant management [15], and when given the choice, many opt for medication or surgical evacuation [12, 13, 16].

At the same time, research suggests that there may be barriers to accessing comprehensive EPL management [17, 18]. Women who seek EPL care at the emergency department (ED) are more likely to be socially and economically vulnerable and are less likely to receive active intervention compared with women who seek care in an ambulatory setting [19]. However, research about miscarriage experiences in the US context has predominantly been conducted with women recruited from healthcare settings [12, 19].

That is, the literature is based predominantly on the experiences of those who have already navigated to an HCP and may fail to adequately capture or illuminate barriers to care.

Taken together, this highlights a key gap in our current understanding: what are the barriers that pregnant people face to receiving comprehensive management for their miscarriage?

### 1.1 Study Context: Indiana

Indiana is a state in the Midwestern US with a population nearing seven million people [20]. The predominantly English‐speaking (89.6%) population is mainly white (83.7%), with Black individuals making up 10.4% and Hispanic or Latino individuals representing 8.8% [20].

Indiana ranks as the 10th unhealthiest state in the nation [21]. It also has the 10th highest maternal mortality rate [22] and 7th highest infant mortality rate [23], among which Black and Brown pregnant people and their children are disproportionately represented [24]. Nearly a quarter of Indiana counties are maternal health deserts without any obstetric providers [25] and the rate of preterm birth is rising [24].

### 1.2 Study Aims

In this qualitative study, we aimed to explore the miscarriage experiences of Indiana residents, identify barriers to high quality patient‐centered care, and generate recommendations to improve comprehensive miscarriage management and information across the state.

## 2 Methods

KL is a medical anthropologist with extensive experience conducting research on abortion and pregnancy. She is a white, cisgender woman and an Assistant Professor of Public Health. AMQ is an Afro‐Caribbean woman, native Spanish‐speaker, and MS student in Public Health. OJF is a Black woman and dual MPH‐PhD student in Public Health, and FL is a Black woman and international PhD student in Public Health. All members of the study team live in Indiana and have resided there for five or more years.

Our project is informed by ethnography as a methodological orientation, with the overall aim of developing a holistic, insider's perspective about the topic [26]. We developed a semi‐structured interview guide specifically for this project based on the literature and KL's extensive research experience interviewing people about their pregnancy outcomes. Two people who had personal experience with miscarriage reviewed the interview guide and provided feedback on the overall tone and flow, as well as the appropriateness of the questions. As a study team, we used an iterative process to refine the interview guide throughout data collection for clarity and flow, but we did not make any major changes. The study instruments are available upon reasonable written request to KL.

KL conceived the idea for the study, led the development of project materials, conducted most of the interviews, and was present for every interview. AMQ led the translation and adaptation of the study materials into Spanish and worked with other Spanish‐speaking Latine students to ensure materials were culturally and linguistically appropriate. AMQ, OJF, and FL contributed to recruitment efforts, and KL and AMQ conducted in‐depth interviews. The Institutional Review Board at Purdue University reviewed and approved this protocol.

### 2.1 Eligibility and Recruitment

Eligible participants were at least 18 years old, sufficiently fluent in English or Spanish to complete the interview, and had experienced at least one miscarriage in Indiana in the preceding five years. We did not have a relationship with participants prior to study commencement, so we used a multi‐modal, community‐based recruitment strategy that included social media posts and advertisements (i.e., Instagram, Facebook), sharing information with community partners across the state, and posting paper fliers.

Our recruitment materials were gender inclusive and we engaged in concerted efforts to recruit a racially and linguistically diverse sample, including developing additional recruitment graphics and posts, and sharing information through our study team's networks and ties to culturally specific organizations. Interested participants were informed that an Assistant Professor in Purdue's Department of Public Health was carrying out the research study “to learn how state‐level laws and policies impact people's experiences with miscarriage to make recommendations for improving care”. To verify eligibility, each person who expressed interest in the study completed a short screening survey before scheduling the interview for a mutually convenient time.

### 2.2 Data Collection

Between June 2023 and July 2024, we conducted 26 interviews either over Zoom or the telephone based on participants' preference. The interviewer conducted the call from a private, locked office, while most participants joined from a private area in their homes. We audio recorded all interviews and participants chose whether they wanted to have video on during the call. Interviews lasted between 50 and 90 min, with most taking about an hour. We reached thematic saturation after approximately 22 interviews and conducted an additional four interviews for confirmation.

Throughout the interview, we encouraged participants to share whatever they felt comfortable with, and we reiterated that participants could end the discussion at any time. We began the interviews by asking about the participant's day‐to‐day life and important relationships. Then, we discussed general health and access to health care before we shifted to talking about the participant's miscarriage experience(s) in depth. This included discussions of how the participant determined she was pregnant, how she determined she was having a miscarriage, and her decision to seek out information from an HCP. We ended the interview by asking participants to reflect on how care and information could be improved and what they wish friends and family understood better. We took notes throughout the interviews and engaged in a process of reflexive memoing [27].

### 2.3 Data Analysis

A team of undergraduate and graduate research assistants (RAs) transcribed the interviews, and each transcript was checked for accuracy by a minimum of two RAs or KL and one RA. KL drew on the study aims, interview notes, and memos to develop an initial code book. Using qualitative software to manage the data, KL carried out content and thematic analysis.

During content analysis, she grouped categories of information and identified recurring patterns in the data [28]. She began by organizing transcripts by type of miscarriage management (expectant, medication, surgical) and then applied codes to track where participants sought information, what they wanted to know, whom they contacted, and where they were referred. This approach helped to effectively trace trajectories of care. She then used Braun and Clarke's [29] phases of thematic analysis to move from describing data to interpreting.

KL identified initial findings and themes and then presented this to the rest of the study team. We engaged in collaborative discussion and refinement of the ideas presented by KL. Then, AMQ, OJF, and FL each reviewed a subset of the transcripts and coded data and looked for negative (or disconfirming) evidence, a deliberate process of examining data that did not fit with expected patterns or theoretical predictions [30]. Throughout data collection and analysis, we met weekly and engaged in an ongoing process of scrutiny and debriefing. We include quotes throughout the Results section to illustrate key points. All names are pseudonyms.

## 3 Results

### 3.1 Participant Characteristics

Twenty‐six English‐speaking, cisgender women who use she/her pronouns, ranging in age from 27 to 46 (mean age: 34), reported on 31 miscarriages that occurred between 5 and 20 weeks’ gestation (mean: 8.5 weeks). Six participants had never given birth, five participants previously had an abortion, and five were pregnant at the time of the interview.

We discussed in‐depth all known miscarriages that happened in the preceding five years. Participants reported on miscarriages that occurred between 2018 and 2023, and most of the pregnancies were desired. Women in our sample had experienced between one and six lifetime pregnancy losses, and no one reported on a miscarriage that took place after Indiana's abortion ban that went into effect in August 2023. The sample was predominantly white (24 white, 2 Latina) and married or in a serious relationship (*n* = 25).

We had respondents from across the state in North, Central, and South Indiana. Although most participants lived in counties that are classified as urban, many participants reported living outside of major cities. We include more details about the sample in Table 1.

**TABLE 1:** Characteristics of participants in a qualitative study on miscarriage experiences in Indiana (*N* = 26).

Characteristics	*n*
Age group (at time of interview) (years)	
18–24	0
25–29	5
30–34	10
35–40	10
41–44	0
45–50	1
Self‐identified race/ethnicity	
White	24
Hispanic/Latina	2
Total pregnancies (lifetime)	
1	2
2	9
3	5
4	7
5 or more	3
Total pregnancy losses (lifetime)	
1	18
2	4
3	1
4	2
5 or more	1
Region of residence	
North	11
Central	10
South	5
County of residence	
Urban	22
Mixed/rural	2
Rural	2

### 3.2 Without Adequate Information, Expectant Management Is a Denial of Care

Almost all the women in our study sought information from a health care provider when they suspected they were having a miscarriage because they lacked knowledge about what to expect during the process and when to seek medical attention. Participants in our study described significant challenges in obtaining clear, actionable information from health care providers about how to manage their miscarriage once they began to experience bleeding, which left many women feeling unsupported and uncertain.

For example, Sophie, a first‐time pregnant woman in her mid‐20s, called her gynecology practice in 2021 after experiencing bleeding at 8 weeks' gestation. She was told, “Unless you're 20 weeks, there's absolutely nothing we can do. Just stay home and wait it out.” Sophie expressed frustration at the lack of guidance, asking: “What about bleeding? What should I be expecting? What should I be looking for? They didn't have a whole lot of information for me.”

When not accompanied by rationale or guidance on what to expect, participants interpreted the recommendation to wait as a denial of care and resources. This lack of communication left participants in our study feeling abandoned as they navigated the physical and emotional challenges of miscarriage while managing work, childcare, and other responsibilities. Kristen said, “I find it quite stressful where you're constantly assessing your own body and how do I feel right now? Is that feeling a thing? Is that something? There's that stress that builds up, and a lot of it for me is just the uncertainty.”

Participants who were instructed to “wait it out” voiced that the advice was incongruent with the realities of their day‐to‐day life. Shannon noted, “I didn't have a choice. It was just wait it out and see what happens. But I do have a life and things like that.” Marissa experienced her miscarriage in 2023 and described the prolonged uncertainty as “almost a month of… never knowing anything for sure, and randomly just waiting to bleed out a bunch of tissue.”

Women felt that the advice they received lacked consideration for their dignity. In Laura's case, the instruction to “wait it out” culminated in her passing pregnancy tissue while she was out to lunch with her friends. “I was so embarrassed because how am I even going to get out of here? I'm in a public place, I'm soaked through my pants, like halfway down my thigh, like soaked through every all my undergarments and my pants [with blood].” Our participants agreed that bleeding sporadically in public places was humiliating and something they wanted to avoid, but the instructions to wait it out did not take this concern into account.

### 3.3 Visiting the Emergency Department for Miscarriage Care Has High Financial and Emotional Costs

The lack of clarity about when and where to obtain care for miscarriage funneled women in our study to the emergency department (ED) either indirectly or through direct referrals from HCPs. When Haley was pregnant in 2023, she did not have a primary care provider and had not yet been able to establish prenatal care. She explained, “I started calling doctors, and I couldn't get in anywhere. I called some walk‐in medical clinics, and they don't do stuff for prenatal care at all. I couldn't find an OBGYN to go to in that short notice. It started scaring me that something was wrong with me, that something was wrong with the baby, so I did eventually go to the emergency room.” Haley was indirectly funneled to the ED because she felt that this was her only option to interact with an HCP.

In other cases, women were instructed directly to seek care at the ED. Nine women in our study tried to obtain care and information for their pregnancy loss in the ED, and most of these nine women were advised by an HCP to go to the ED. Six other women were directed to go to the ED but decided against it due to what they anticipated would be a significant out‐of‐pocket cost.

Lauren experienced her miscarriage in 2022 when she was in her early 30s. She started bleeding at home and then explained, “I called my gynecologist to see what are the next steps, what do I do? They told me that I had to go the emergency room. There was no other option. They wouldn't see me, just wanted me to go to the ER.” She continued, “I was honestly so mad …. [They] asked me about my pain level, and then immediately said: just go to the emergency room. Is that all you know? I don't want that.” Lauren's story exemplifies a disconnect between the kind of information and care she was seeking and what her HCP recommended.

Like Lauren, several of our participants were instructed to go to the ED based on their pain level but reported that this was vague and challenging to interpret because “everybody's pain is different”. Shannon said, “They told me, ‘Oh, well, if the pain gets really bad, go to the ER.’ That's very vague.” Once participants arrived at the ED, most providers did not offer pain medication or discuss strategies for pain management. This created confusion and exacerbated frustrations about what participants described as an overall low quality of care for pregnancy loss in the ED.

Participants who obtained care for pregnancy loss in the ED unanimously reflected on the experience as negative, often costly, and in some cases, “traumatizing”. Shannon said succinctly, “If I hadn't gone to the emergency room, [my miscarriage] would not have been nearly this expensive or nearly this stressful.” Women perceived health care providers in the ED as lacking compassion and uncomfortable with treating and discussing pregnancy loss. Alexis had a miscarriage in 2019 and brought up her dissatisfaction with the quality of care she received with a nurse at the ED. In response, “The nurse said, ‘They are emergency room doctors. They're not comfortable with talking about that stuff. They just aren't equipped to have those conversations’.”

None of the women in our study who went to the ED were offered medication or surgical management. Shannon said, “I would have taken them up on something to induce it. When I first went in, they were like, ‘Oh, you're through the worst of it.’ And I hadn't even started [bleeding], really.” Maria, a Latina woman who had a miscarriage in 2021 and went to the ED when she started bleeding, was informed that a D&C was not an option for her. “They weren't going to do a D&C because they didn't want to be invasive. They were like, your body is just going to do what it's going to do. But they didn't tell me what to expect.” In this case, Maria would have preferred to have a surgical evacuation to have more control over her miscarriage. She explained that she still does not understand why she could not have the procedure she wanted.

A few participants were set up with serial blood draws to track beta‐hCG levels and determine the status of their pregnancy, but generally participants reported that once they arrived at the ED, they were told to go home and consult with an OB‐GYN. Since many of the women who arrived at the ED were instructed to go there by another healthcare provider, this experience was frustrating and confusing.

### 3.4 Providers' Approaches to Discussing Intervention Constrained Patient Choice

In our study, most of our participants did not report that they were offered treatment options for miscarriage. Conversely, only participants who discovered their pregnancy loss during a routine prenatal care appointment were offered active intervention. However, many participants reported that healthcare providers delivered the news that the pregnancy was not viable and then immediately began discussing treatment options. Participants characterized this as poor bedside manner and described an informational and emotional overload that made it difficult to meaningfully engage with the information being presented, ask questions, or raise concerns. Samantha had a miscarriage in 2021 and explained, “[The doctor] launched right into treatment options and I wasn't able to ask any questions about the details or really make sense of the options in that moment, because I was still processing the fact that I was having a miscarriage.”

Angela described a similar experience when she had a miscarriage in 2023. “While the [transvaginal] ultrasound is happening, the OB is talking about, ‘You could take a pill, or you can have a procedure.’ And I'm like, ‘Can you get out of my vagina and let me think?’ My husband's crying, I'm crying’.”

Megan was the only woman that we spoke with who declined the offer of an intervention in favor of expectant management. In hindsight, she is unsure whether this was the best decision for her, as she reflected “I didn't get a D&C. I didn't take any medicine. And looking back, I wish I had. That was the worst.” However, she did not have a solid understanding of what was involved in the procedure. Her HCP did not offer clarifying information, and she did not feel like she could ask questions or express uncertainty. She explained, “[The doctor] said I could do a D&C, which is basically like an abortion right? They go in and kind of scrape it? I didn't express this to him, but I was afraid that it would screw up my body so that I would not be able to get pregnant again. That was a concern that I had that. I was afraid they would scrape a part of me off that I was going to need later.”

## 4 Discussion

The need to improve the quality of miscarriage care in the ED is well documented [19, 31, 32, 33], but the pathways through which patients arrive at the ED when experiencing pregnancy loss have not been well researched [34]. Our study begins to address this gap and suggests there are several actionable strategies that could improve the provision of EPL care inside and outside of the emergency department across Indiana. To address the unnecessary referral of patients to the ED, major gaps in outpatient support, and the significant emotional and financial strain caused by current protocols and pathways to care, we recommend a comprehensive approach involving healthcare systems, providers, and community members. Leveraging online spaces for social support around pregnancy loss could also be a promising initiative [35].

ACOG recommends that pregnant people contact their gynecologic provider if they experience vaginal bleeding [1]. Many participants followed this recommendation and were frustrated with the result. Indeed, some women in our study reported arriving at the ED because they were referred there by HCPs, indicating that a proportion of patients could be more appropriately diverted elsewhere. It is possible that referrals to the emergency department represent an overcorrection by the health system in response to persistently high maternal mortality rates across the state. However, unnecessary referrals contribute to ED overcrowding, which has been shown to negatively affect the quality of care provided and increase the risk of adverse outcomes for patients [36]. Future research should investigate referral practices for vaginal bleeding across various types of health care providers that care for pregnant people (e.g., general practitioners, OB‐GYNs, midwives) with a focus on understanding the factors that drive these decisions. This includes better understanding why women across the state report being turned away from, and denied, care.

Indiana faces a shortage of obstetricians and birth workers, which contributes to inadequate prenatal care for many [25, 37]. Beyond this, the timing of EPL often results in miscarriages occurring before individuals can establish prenatal care [1]. Although this dynamic is exacerbated by the provider shortage, it also emphasizes the need for interventions to address the lack of information and support for miscarriage outside of gynecologic settings. Many family medicine residency programs do not routinely train clinicians in early abortion care or miscarriage management, despite these falling within their scope of practice [38]. Programs like the Miscarriage Care Initiative work to integrate comprehensive EPL into primary care and have been shown to expand access [39], which could serve as a valuable model for Indiana. Efforts should also focus on educating prenatal care providers, such as obstetricians and midwives, about the realities of patient experiences in the ED.

Yet, patients experiencing vaginal bleeding and miscarriage will inevitably continue to seek care at the ED. Our findings reinforce the need to strengthen the provision of EPL care in the emergency setting. This includes ensuring ED providers are equipped with knowledge of appropriate treatment options, including how to offer expectant, medication, or surgical management when appropriate [40]. The development and use of established outpatient protocols may support these efforts. Additionally, training on how to counsel patients experiencing pregnancy loss with sensitivity and clarity could improve patient experiences and outcomes. Even if access to active interventions remains limited, providing patients with appropriate information to support expectant management would be a meaningful improvement.

### 4.1 What's Next for Indiana?

Following the 2022 *Dobbs v. Jackson Women's Health* decision, physicians and advocates have raised concerns that amidst increasing restrictions on abortion care across the United States [41], treatment options for miscarriage care will be negatively impacted [42, 43]. In Catholic hospitals that prohibit abortion due to religious doctrine, miscarriage care has been significantly delayed or even denied due to clinicians' lack of clarity about the circumstances that classify a procedure as either a miscarriage or an abortion [44].

Yet, our study suggests that a proportion of women in Indiana were extremely constrained to find information about, and ways to manage, their miscarriage even before abortion was banned across the state. As of August 2023, abortion is banned in Indiana with limited exceptions for pregnancies that resulted from rape or incest (up to 12 weeks') or lethal fetal anomaly (up to 22 weeks') [45]. Very few legal abortions occur in the state [46] but in January 2025, Indiana legislators introduced Senate Bill 171 (SB 171), which would have banned the use, prescription, and possession of abortion‐inducing drugs under all circumstances, without exception [47]. This legislation would prevent clinicians from providing evidence‐based treatment options consistent with the standard of care and would increase risks for individuals experiencing miscarriage. Although the bill did not pass, it highlights a broader legislative trend toward restricting access to medications commonly used in both abortion and miscarriage care and underscores the need to address both together in policy discussions.

Abortion restrictions impact clinicians' ability to provide appropriate care [44, 48] as well as medical students' decisions about where to apply for residency [49] and the broader HCP workforce [50, 51]. Indiana already has a documented shortage of providers, and the true impact of the state's abortion ban on the medical workforce has yet to be seen. That Indiana women are already suffering from a lack of access to comprehensive pregnancy care and treatment options for early pregnancy loss is concerning and warrants urgent intervention across the state.

## 5 Limitations

Despite our efforts to recruit a racially and linguistically diverse sample, the participants in our study were predominantly white and English‐speaking. As well, most women talked about losing desired pregnancies. Not all people who experience pregnancy loss feel this way about their miscarriage, and this may influence their desire for, or trajectory to, care. In‐depth interviews are subject to participants' recall bias, but we made efforts to mitigate this by focusing on miscarriages that occurred in the last 5 years.

## Funding

This work was supported by the Indiana Clinical and Translational Sciences Institute, Purdue Women's Global Health Institute, Purdue University, and National Institutes of Health.

## Conflicts of Interest

The authors declare no conflicts of interest.

## Data Availability

The data that support the findings of this study are available from the corresponding author upon reasonable request.

## Endnotes

^1^
People of all genders can, and do, experience pregnancy loss. Throughout this manuscript, we prioritize the use of language that is both inclusive and accurate to the data that were collected. When describing prior research, we use the terminology of the original authors.
